# Mechanism of sodium chloride in promoting reduction of high-magnesium low-nickel oxide ore

**DOI:** 10.1038/srep29061

**Published:** 2016-07-04

**Authors:** Shiwei Zhou, Yonggang Wei, Bo Li, Hua Wang, Baozhong Ma, Chengyan Wang

**Affiliations:** 1 State Key Laboratory of Complex Nonferrous Metal Resources Clean Utilization, Kunming University of Science and Technology, Kunming 650093, China; 2 School of Metallurgical and Ecological Engineering, University of Science and Technology Beijing, Beijing 100083, China

## Abstract

Sodium chloride has been proved that it is an effective promoter for the reduction of high-magnesium, low-nickel oxide ore. The aim of current work is to clarify the promotion behavior of sodium chloride in the roasting reduction process. The influence of moisture on the reduction of ore in the presence of sodium chloride is studied to get clear comprehension of promotion process. In the presence of moisture, the HCl is produced by pyrohydrolysis of sodium chloride for chlorinating nickel and iron oxides, moreover, interactions between metallic oxides and sodium chloride are also a way for chlorination at high temperature (>802 °C); subsequently, the metal chloride would be reduced by reductant. In the absence of moisture, the magnetic separation results show that the recoveries of iron and nickel have a significant increase; moreover, olivine structure would be destroyed gradually with the increase of roasting temperature in the action of sodium chloride, and the sodium chloride existed in high-magnesium, low-nickel oxide ore could make the NiO isolate from NiO-bearing minerals. The NiO reacts with Fe_2_O_3_ at high temperature to form NiFe_2_O_4_, which is conductive to the formation of Ni-Fe alloy during the reduction process.

Nickel is an important strategic alloying metal due to its special physical and chemical characteristics. The continued depletion of tractable high grade nickel sulfide ore have led to greater efforts to extract nickel from laterite ore^1^,^2^. Nickel laterites, which contain ~60% of the world’s total landbased nickel resources, account for ~40% of annual global nickel production on the basis of literature reported^3^. Nickel is mainly hosted in a variety of hydrous Mg silicates and smectites within laterite ore in the form of isomorphism and/or adsorption. There isn’t, therefore, a unified treatment method for utilization of nickeliferous laterite ores.

Laterite ore processing methods are classified on the basis of their chemical compositions. The limonitic ores, which contain typically 0.5–1.7% nickel and 40–60% iron^4^, are suitable for a hydrometallurgical process, in particular, High Pressure Acid Leaching (HPAL)^5^,^6^. The saprolitic ore deposit is rich in magnesium silicates and nickel content (1.5–3%). This ore deposit, therefore, is more amenable to treatment by pyrometallurgical techniques. However, the conventional pyrometallurgical processes are typically suitable for process laterite ore with nickel content over 1.5%^7^, such as rotary kilns-electric furnaces (RKEF) and Krupp-Renn processes^4^,^8^,^9^,^10^. The low nickel laterite ores, which nickel grade less than 1.5%, widely exists in the nature; consequently, the process of roasting reduction followed by magnetic separation has been proposed to obtain ferronickel concentrate from low grade (<1.5% Ni) nickel laterite ore^11^,^12^. The key of this process is to add a suitable additive for promoting the reduction of ore.

According to the reported studies, sulfocompound is the most commonly additives, the mainly functions may be summarized as follow: (a) the sulphur additive can suppress the formation of fosterite phase when laterite ores are calcined; (b) S can promote the grain growth of nickel and iron, so as to improve the Ni grade and recovery^12^,^13^,^14^,^15^. A ferronickel concentrate containing 6.00% Ni, with a nickel recovery of 92.10%, can be obtained from the nickel laterite ore (1.42% Ni), the reduction was carried out at 1100 °C for 60 min, with the addition of 6% calcium sulfate and 5% coal^7^. In addition, when the laterite ore (1.38% Ni) was roasted at 800 °C for 220 min at a total gas flow rate of 200 L/h (H_2_: 70%, N_2_: 30%), with the addition of 20% sodium sulfate, a ferronickel concentrate with 5.63% Ni can be obtained, and the corresponding recovery is 83.59%^15^. Nevertheless, complex desulfurization process and the formation of a low-melting-point sulfur compound (FeS) in the steel contributed to their difficult development.

Chlorine salt is another effective accelerant on the basis of reported literatures. By the middle of sixteenth century, the salt was added into the leaching process of ore to improve the effect of extracting silver with the method of amalgamation^16^. Chlorides have the characteristics of low melting point and volatile, the chlorination and evaporation method are generally used for extracting metal from ore and/or impurity elimination^17^,^18^,^19^. More recently, magnesium chloride has been used as a chloride agent to extract Ni and Co from low-grade nickel laterite, the results show that a concentrate containing 5.25% Ni can be obtained, the corresponding recovery is 91.5%^20^. Similarly, the sodium chloride has been used for enrichment of ferronickel concentrate from low nickel laterite ore by our group before^21^. The experimental results indicate that a ferronickel concentrate with 7.09% Ni and 67.90% Fe can be obtained from the high-magnesium low-nickel (HL) oxide ore containing 0.82% Ni and 9.67% Fe via chloridization and reduction roasting at 1200 °C for 20 min in the presence of 10 wt.% sodium chloride and 8 wt.% coal, and the corresponding recoveries of nickel and iron were 98.31 and 72.08%, respectively. Compared with the results of aforementioned studies, as an effective accelerator to promote the reduction of low grade nickel laterite ore (<1.5% Ni), the advantages of sodium chloride are mainly reflected in two aspects: (a) in the presence of sodium chloride, the roasting duration is shorten (b) and the recoveries of Ni and Fe are higher than the additives of sulfocompound. Furthermore, sodium chloride could also avoid the adverse effects of quality steel product.

Although the mechanism of promoting the reduction of ore by chloride salt has been studied in the literature^20^,^22^,^23^, there is a paucity of information available on the effect of moisture on reduction of ore. Additionally, in many of the relevant mechanistic studies, moisture, which is required for the production of hydrochloric acid, has been considered to be an essential component in the process of chlorination. However, it is known that the hydrochloric acid existed in system is serious harmful to the metallurgical equipment. Consequently, in the current work, the moisture within ore system is taken into account to avoid the generation of hydrochloric acid and a detailed mechanistic analysis is performed of the chloridization and reduction roasting of HL oxide ore in the presence of sodium chloride. The effect of moisture on the grades and recoveries of Ni and Fe within concentrate is determined. Phase transition of iron nickel oxides and the microstructure change of HL oxide ore during the chloridization process are also studied to analysis the behavior and effects of sodium chloride within ore in the absence of moisture. Where possible, the results are a comprehensive supplement to the mechanism of sodium chloride within ore.

## Experimental Section

### Materials

The laterite ore sample used in this study came from Yunnan province of China; it belongs to a special kind of laterite nickel ore, which contains approximately 31.49% of magnesium and 0.82% of nickel (Table 1). A low cost anthracite (76.43% of fixed carbon content) was used for reducing laterite ore. The sodium chloride used in this study was of chemical grade.

### Apparatus and procedure

A series of roasting tests were performed in a horizontal tube furnace using corundum crucibles. First, a dried ore sample with 10 wt.% sodium chloride and/or 8 wt.% anthracite was homogeneously mixed in a laboratory rotary mixing drum and transferred into the corundum crucible. Then, the corundum crucible was placed in the constant-temperature zone of the tube furnace and heated to the required temperature at a constant rate of 10 K min^−1^. After calcination, the roasted ore was cooled under nitrogen atmosphere in the tube furnace to prevent re-oxidation. A sub-sample of the cooled pellets was ground with a small amount of water by a vibratory mill to 98% below 75. The slurry was then separated in Davies Magnetic Tube (wet magnetic separation) using a magnetic field intensity of 150 mT.

Magnetic separator used in this study was a wet magnetic separator, a commercial product of TDT Ltd., China. The principle of magnetic tube method is based on reciprocating and rotating pendulum movement made by a glass tube installed between two poles of electromagnet. When the slurry in the magnetic tube is passing the magnetic field, the nonmagnetic particles in the mechanical movement will be washed by water and discharged. While the magnetic particles will be attached on the wall of tube and flushed into a collecting bin to obtain Ni and Fe concentrate, after the magnetic field was turned off.

The compositions of concentrate were analyzed by chemical analysis, and the recoveries of Ni and Fe were calculated by the following formula:


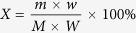


where *X* is the recovery of nickel or iron; *m* is the weight of concentrate, *w* is the nickel or iron content in the concentrates, and *M* is the weight of raw ore, *W* is the nickel or iron content in the raw ore.

### Analysis method

Thermogravimetric (TG) and differential scanning calorimetry (DSC) analysis were performed on a NETZSCH STA 449F3 unit under a nitrogen atmosphere, at a heating rate of 10 K min^−1^. The ore pore structure and specific surface area were analyzed by nitrogen adsorption method using Quantanchrome Instruments CHEM BET-3000 equipment. X-ray diffraction (XRD) experiments were performed on a Japan Science D/max-R diffractometer. The microstructures and modes of occurrence of the main elements of the roasted ores were analysed by scanning electron microscopy (SEM; HITACHI-S3400N) equipped with energy dispersive X-ray spectroscopy (EDS). Materials for SEM examinations were prepared using standard resins mosaic and polishing techniques.

## Results and Discussion

### Effect of moisture on beneficiation of Ni and Fe

**Figure 1:**
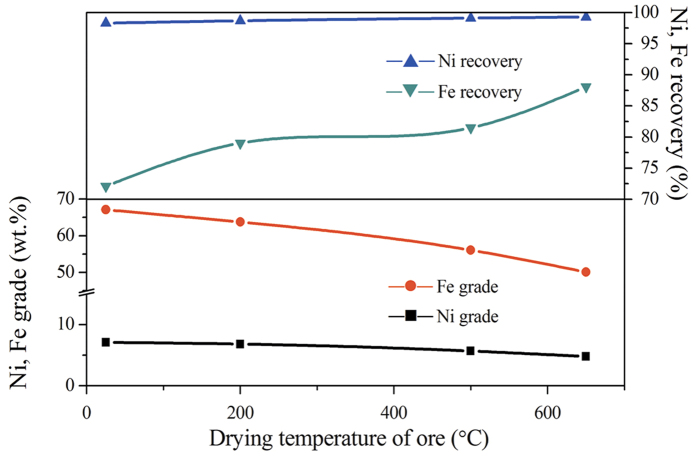
Effect of drying temperature on the grades and recoveries of Ni and Fe.

Figure 1 shows the magnetic separation results of HL oxide ore roasted with 10 wt.% NaCl and 8 wt.% anthracite at 1200 °C for 20 min as a function of drying temperature (T_D_) of raw ore. The both grades of Ni and Fe decrease from 7.09 and 67.09% to 4.79 and 50.09% with increasing T_D_, whereas the corresponding recoveries are increased; in particular, the recovery of Fe has a significant increase, which increased from 72.08% to 88.06%. The more iron is recovered, the grade of the resulting ferronickel is lower^4^; thus, the grades of Ni and Fe have a decrease.

**Figure 2:**
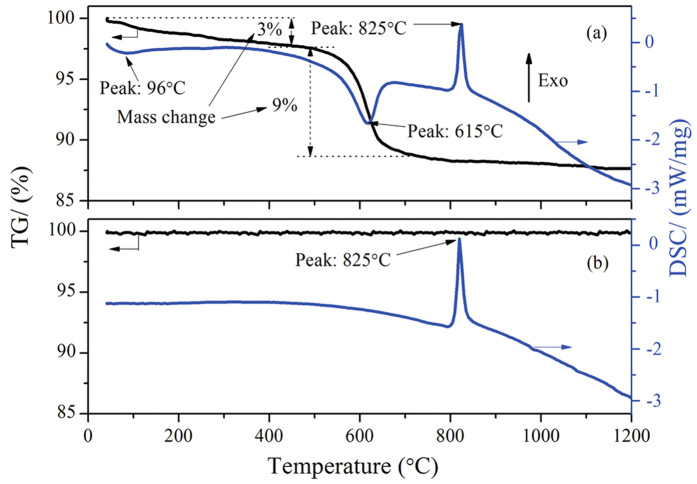
TG/DSC images of HL oxide ore ((a) raw ore; (b) dried at 650 °C).

**Figure 3:**
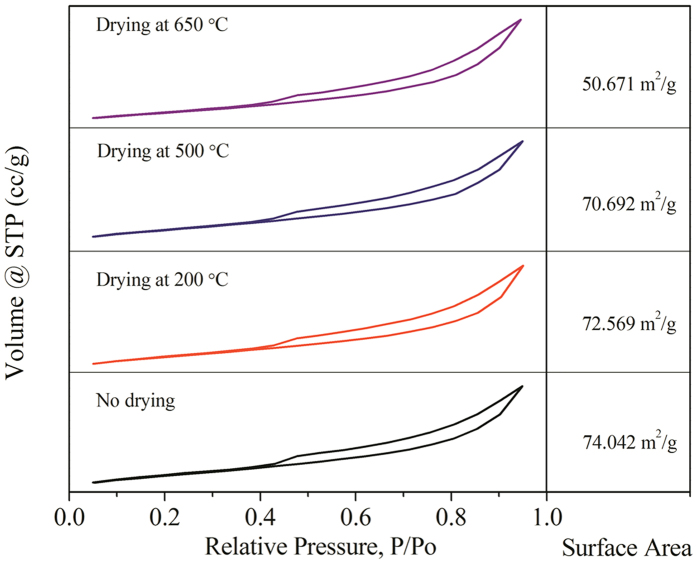
Pore structure and specific surface area of HL oxide ore as a function of drying temperature.

To clarify the ore changes after roasting at various T_D_, TG/DSC and surface area of raw ore are conducted and respectively show in Figs 2 and 3. It can be seen from Fig. 2a that raw ore mainly contains ~12% of moisture. Free water will evaporate at 100 °C with a weakly endothermic peak; while the crystallization water combines with lizardite and completely releases at around 615 °C (Fig. 2a); moreover, crystallization of anhydrate silicate may form olivine group at around 825 °C with a strong exothermic peak^24^,^25^. After roasting at 650 °C, the mass of ore remains unchanged and the endothermic peak around 615 °C disappears as shown in Fig. 2b, this finding indicates that the moisture is completely removed. In addition, there is not apparent change about pore structure of ore with the increase of T_D_ from room temperature to 650 °C (Fig. 3), while the specific surface area decreases gradually as T_D_ increased. When the T_D_ increases from 500 to 650 °C, the specific surface area decreases from 70.692 to 50.671 m^2^/g sharply.

As analyzed above, the main changes of the ore after roasting at 650 °C are that the moisture is completely removed and the specific surface area decreased. Decrease of the specific surface area is not conducive to the reduction of ore in gas-solid reaction process; furthermore, the overall reducibility of laterite ore may decrease with increasing roasting temperature due to the formation of irreducible phases^26^. Nevertheless, the magnetic separation results (Fig. 1) indicate that the reduction of iron and nickel oxides increases as T_D_ increased on the basis of both recoveries. This implies that upgradation of chloridization and reduction may be strongly affected by reducing moisture. Based on the literatures^22^,^23^, chloridization and segregation are mainly carried out in the presence of moisture during reduction roasting process. In the absence of moisture, however, the NaCl can also promote the HL oxide ore reduction (Fig. 1). This phenomenon has not been reported in the existing literature yet. For this reason, the subsequent discussions mainly considers T_D_ = 650 °C.

### Effect of NaCl on chloridization behavior in HL oxide ore

In the presence of moisture, nickel and iron oxides are mainly chlorinated by hydrogen chloride, which is produced by pyrohydrolysis of sodium chloride under the catalysis of silica^23^; the process is based on the reactions (1) to (3). Simultaneously, interaction of solid chloride agent with iron and nickel oxides within ore may be also a way for chlorinating metal oxides. At high temperature (>801 °C), sodium chloride in the molten state is able to make full contact with the components in ore; as raising temperature higher, the volatilization of chlorination products (NiCl_2_, FeCl_2_) increased so that the equilibrium of reactions (4) - (5) shifts to positive; furthermore, sodium oxide, which is generated by sodium chloride, reacted with SiO_2_ and/or Al_2_O_3_ to form complex compound [reaction (6)], thus, the activity of sodium oxide within system could be decreased.


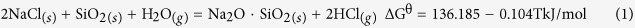



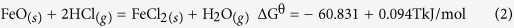



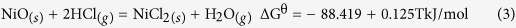



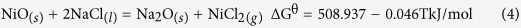



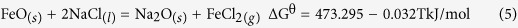



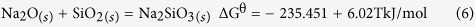


**Figure 4:**
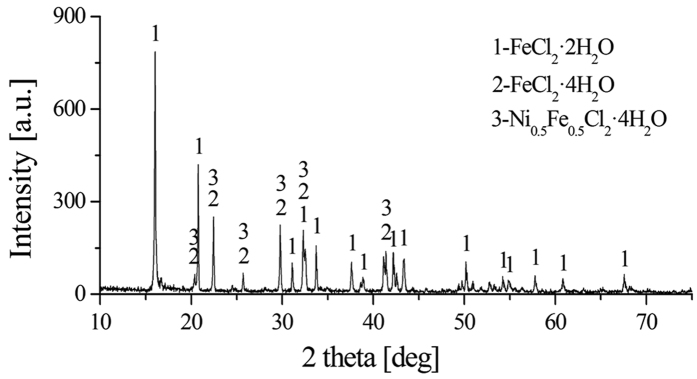
XRD pattern of volatile matter.

An experiment is performed under the roasting conditions (HL oxide ore dried at 650 °C, continuous heating up to 1200 °C, 10 wt.% sodium chloride dosage) to collect the volatile matter. The XRD result of the volatile matter (Fig. 4) shows that the main phases are rokuhnite [FeCl_2_·2H_2_O], iron chloride hydrate [FeCl_2_·4H_2_O] and iron nickel chloride hydrate [Ni_0.5_Fe_0.5_Cl_2_·4H_2_O]. It can be seen from Fig 2b, there is no moisture within HL oxide ore after drying at 650 °C. Thus, the crystalline water in volatile matter may be attributed to the moisture in air. Ferrous chloride, which is an easily deliquescent compound, is in inevitably contact with air and absorbed its moisture to form crystalline hydrate. Nonetheless the interactions are indirectly certified by the XRD result (Fig. 4), they are not the main way for sodium chloride to promote the reduction of laterite ore. From a kinetic viewpoint, the interaction rate between solid-solid/liquid phases would be limited due to the poor contact of their phases^27^. The followings are the relevant equations of chlorides deliquesce:


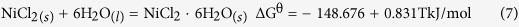



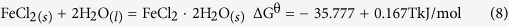



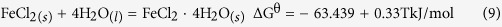


### Phase transformation of HL oxide ore during roasting process

#### TG/DSC of HL oxide ore in the presence of sodium chloride

**Figure 5:**
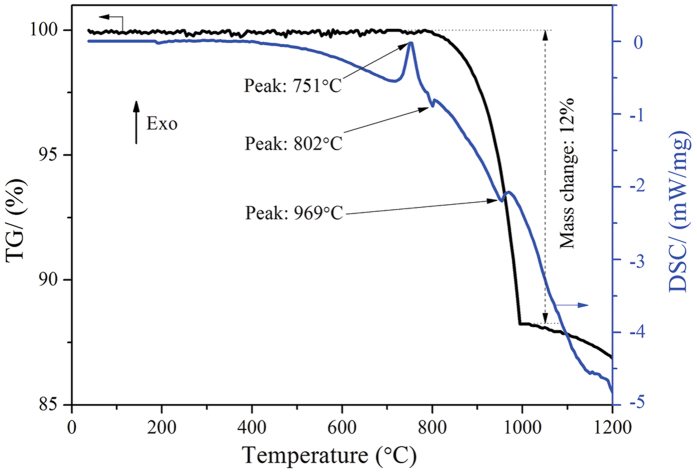
TG/DSC of dried HL oxide ore in the presence of sodium chloride.

**Figure 6:**
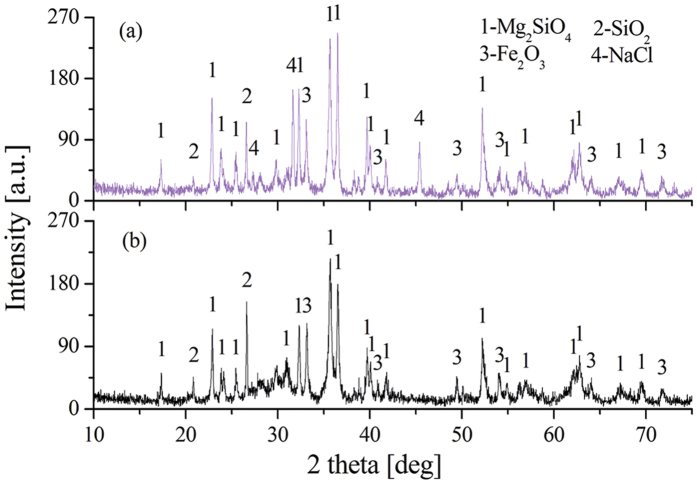
XRD pattern of roasted ore under two conditions ((a) roasted at 751 °C with NaCl; (b) roasted at 825 °C without NaCl).

In the previous section, NaCl has been proven to promote the reduction of HL oxide ore in the absence of moisture. Interactions would not be the main route for promoting ore reduction because of the kinetic conditions. Thus, a series of experiments are performed to investigate the main behavior of NaCl in dried HL oxide ore system. Figure 5 depicts the TG/DSC result of laterite ore (dried at 650 °C) with 10 wt.% NaCl. In contrast with Fig. 2b, in addition to mass loss, the DSC curve is great different in the aspect of peak position. Two new weak endothermic peaks appeared in Fig. 5: the first weak endothermic peak corresponds to a temperature of 802 °C and is mainly attributed to the melting of sodium chloride; the second endothermic peak corresponds to a temperature of 969 °C and is mainly attributed to the volatilization of chlorides. Moreover, compared with Fig. 2b (825 °C), the position of exothermic peak in Fig. 5 (751 °C) shifts to low temperature direction. Subsequently, Fig. 6 shows the XRD results of dried ore roasting at 751 °C (with NaCl) and 825 °C (without NaCl). In both conditions, the XRD patterns show Mg_2_SiO_4_ as the major phase, with SiO_2_ and Fe_2_O_3_ as minor phases. Fe_2_O_3_ peak intensity has no obvious distinction, while the presence of NaCl increased peak intensity of Mg_2_SiO_4_ and reduced the peak intensity of SiO_2_ in Fig. 6. This trend indicates that NaCl can promote the formation of olivine group from the crystallization of anhydrate lizardite. The reason may be that the Cl^−^ could intrude into the grain boundary of serpentine^28^, the Si-O bond is prior fractured by this exogenic action as a result. Thus, sodium chloride can lower the reaction temperature, and shorten the reaction time to improve efficiency. Furthermore, Mg_2_SiO_4_ generated in advance benefits the formation of MgSiO_3_ on the basis of reaction (10) as reduction temperature increased. NiO embedded in MgSiO_3_ is easier to reduce than that in Mg_2_SiO_4_^26^.


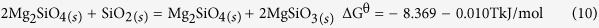


#### Phase transformation of HL oxide ore in the absence of sodium chloride

**Figure 7:**
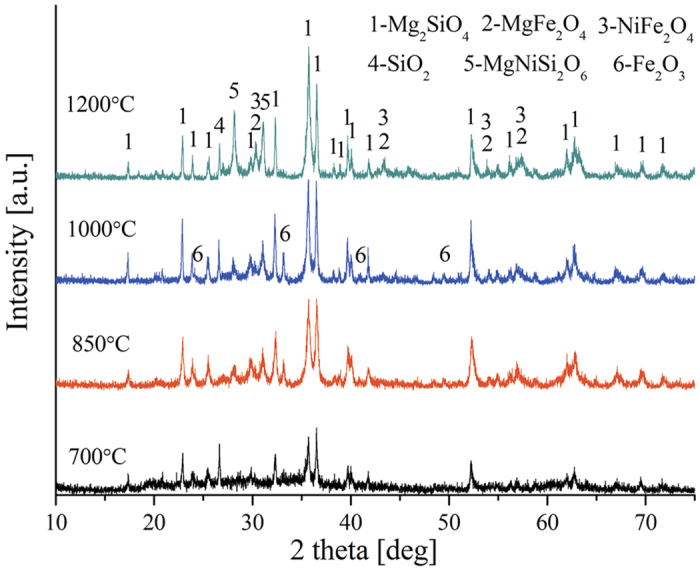
XRD patterns of HL oxide ore roasted at various temperature.

The phase transformations occurring during heat treatment (700 to 1200 °C) of dried raw ore are shown in Fig. 7. The main minerals, which are evident from the X-ray diffraction patterns of the HL oxide ore, are forsterite [Mg_2_SiO_4_] and hematite [Fe_2_O_3_] and the minor phase are spinel, magnesium nickel silicate [MgNiSi_2_O_6_] and quartz [SiO_2_]. As temperature is increased to 850 °C, the hematite peaks begin to appear and the intensity increases with increasing roasting temperature. At 1200 °C, the presence of spinel, including MgFe_2_O_4_ and NiFe_2_O_4_, becomes obvious with formation of peaks reflecting this mineral phase, on the contrary, the hematite peaks have completely disappeared. This may be due to the reactions (11) - (12) occurring at high temperature.


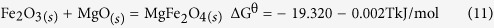



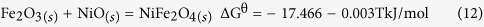


Moreover, magnesium nickel silicate [MgNiSi_2_O_6_] peaks become more dominant as temperature increased to 1200 °C. This result indicates that the higher roasting temperature would not be advantageous for the aggregation of nickel and iron so long as roasting temperature below the melting temperature of HL laterite ore.

#### Phase transformation of HL oxide ore in the presence of sodium chloride

**Figure 8:**
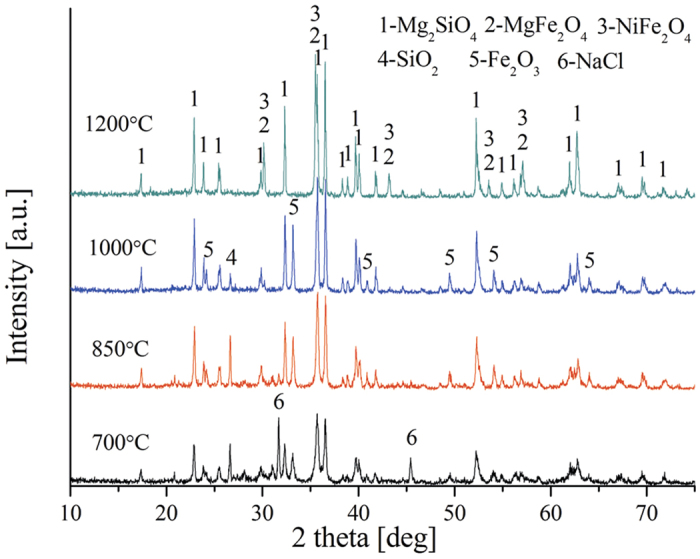
XRD patterns of HL oxide ore in the presence of NaCl roasted at various temperature.

Effect of NaCl on the transformation behavior of dried raw ore during roasting at high temperature are shown in Fig. 8. The X-ray diffraction patterns of the samples show forsterite [Mg_2_SiO_4_] as the major phase, with hematite [Fe_2_O_3_], spinel and quartz [SiO_2_] as minor phases. The forsterite diffraction peaks increase with increasing roasting temperature. However, the Fe_2_O_3_ diffraction peaks increase with the increase of roasting temperature from 700 to 1000 °C, and then completely disappeared; this phenomenon is in accordance with the previous results in Fig. 7. The diffraction peaks of spinel group (MgFe_2_O_4_ and NiFe_2_O_4_) begin to appear at 1000 °C and become dominant as temperature increased to 1200 °C. Sodium chloride peaks exist in the Fig. 8 at 700 °C, and disappeared gradually after 850 °C.

**Figure 9:**
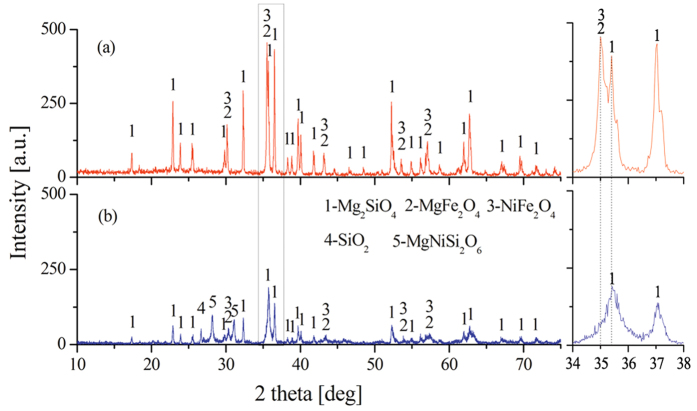
XRD patterns of HL oxide ore roasted at 1200 °C ((a) with NaCl; (b) without NaCl).

In the absence and presence of sodium chloride, the XRD patterns of laterite ore roasted at 1200 °C are presented in Fig. 9. In the action of sodium chloride, the diffraction peaks intensity of forsterite and spinel increased significantly and the diffraction peaks of magnesium nickel silicate [MgNiSi_2_O_6_] disappeared. This finding demonstrates that the sodium chloride could obviously promote the decomposition of magnesium nickel silicate into serpentine and nickel oxide upon equation (13). The NiO, which separated from serpentine, reacts with Fe_2_O_3_ to form NiFe_2_O_4_ so as to increase the diffraction peak intensity of spinel, namely, the formation of NiFe_2_O_4_ strengthens the aggregation of nickel and iron.


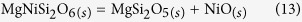


### SEM analysis of HL oxide ore

**Figure 10:**
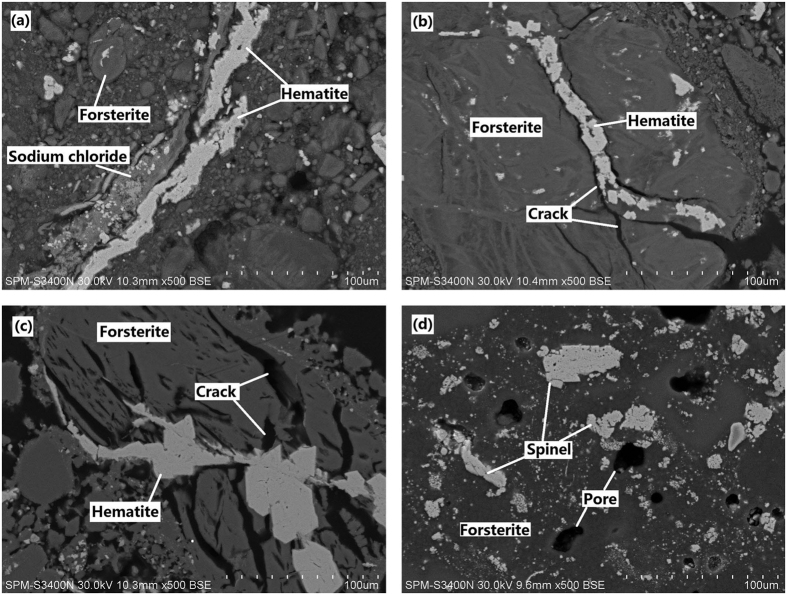
SEM images of HL oxide ore with NaCl roasted at various temperature ((a) 700 °C; (b) 850 °C; (c) 1000 °C; (d) 1200 °C).

Figure 10 shows the microstructure of HL oxide ore roasting at various temperature (700 to 1200 °C) in the presence of sodium chloride, the mode of occurrence of main elements is analyzed by EDS. The white grey and charcoal grey substances observed in Fig. 10a–c are iron oxide and forsterite, respectively; however, the white grey particle in Fig. 10d is spinel, which contains NiFe_2_O_4_. The sodium chloride phase is observed and adjacent to the iron oxide phases, as shown in Fig. 10a. This finding indicate that with the action of sodium chloride, the aggregation of iron oxide occurred during heating process. As roasting temperature is increased to 850 °C, the NaCl intrudes into ore and hard to observe its phase in Fig. 10b, meanwhile, the iron oxide bearing forsterite is damaged and the iron oxides aggregates to the cracks of forsterite. When the roasting temperature is increased to 1000 °C, the structure of forsterite is seriously damaged and the iron oxides particles further grow up (Fig. 10c). Further increasing the temperature to 1200 °C, the HL oxide ore sinters and many small pores appears inside the ore; the spinel particles generate and aggregate to form large particles (Fig. 10d).

As stated above, the sodium chloride and iron oxide coexisted within ore without the structural failure of olivine before the melting point temperature of sodium chloride. However, after the melting state of sodium chloride, the structures of olivine have a significantly change, which is destroyed gradually with increasing temperature. In terms of crystal structure, the thermodynamic properties and molecular reaction dynamics of melted sodium chloride, such as density, Madelung constant, volume thermal expansion coefficient and diffusion coefficient, varies with temperature. As a result of the Madelung constant increases with increasing temperature, the disorder degree about the melted NaCl system becomes more dominant lead to an unstable crystal structure of sodium chloride^29^; in addition, the diffusion coefficient is proportional to temperature and the density of NaCl decreases with increasing temperature. Thus, the diffusion ability of NaCl strengthened at high temperature and more easily infiltrates into the grain boundary of olivine. Furthermore, at high temperature, the volume thermal expansion coefficient of melted NaCl also increases with increasing temperature gradually^30^,^31^,^32^; this molecular reaction dynamics may be the main reason of the destruction of olivine structure (Fig. 10). Figure 10d clearly shows the compact structure of olivine roasted at 1200 °C. In the case of the roasting at 1200 °C, the NaCl provides a liquid phase environment to promote the sintering of forsterite; this is in good accordance with the results for the study^33^.

**Figure 11:**
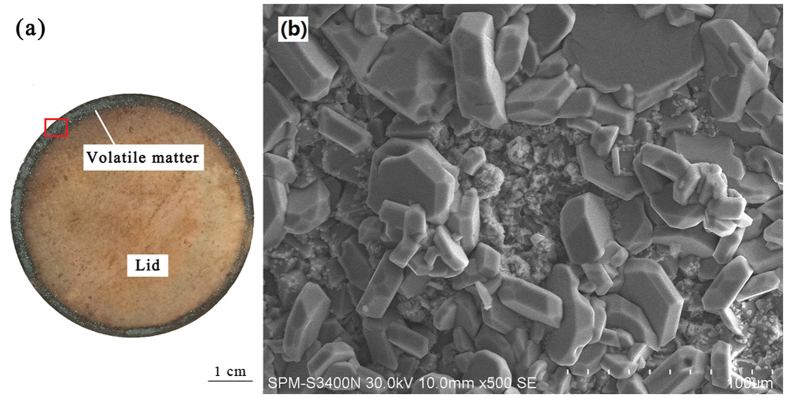
SEM image of volatile matter on the lid.

**Figure 12:**
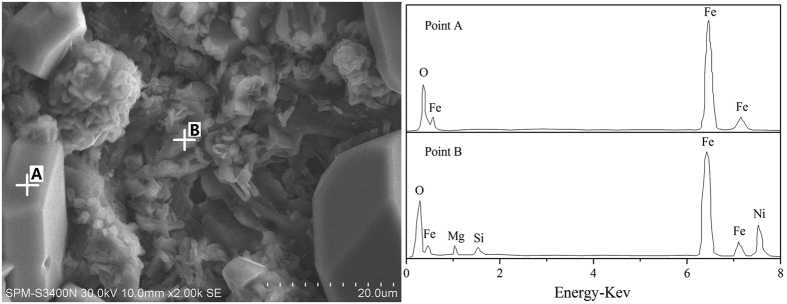
SEM/EDS analyses of volatile matter on the lid.

The pores within ore (Fig. 10d) indicate that gas phases evaporated from the ore system. To verify the compositions of volatile matter, a chloridizing roasting test, which is put into corundum crucible with a lid, is carried out, then analyze the volatile matter on the lid by SEM-EDS. For volatile matter, the crucible lid photo is shown in Fig. 11a, and its SEM images show that two different morphologies (massive and flocculent) existed in the volatile matter (Fig. 11b). Based on the EDS analyses (Fig. 12), the composition of massive substance is relatively simple and is iron oxides; the flocculent substance contained complicated compositions, the oxygen, iron and nickel are the main elements, the magnesium and silicon are the minor elements. Thus, the main compositions within flocculent substance may be nickel and iron oxides. The formation of volatile matter may be mainly attributed to the following two reasons: (1) molten sodium chloride gathered together with nickel and/or iron oxide monomer, the volatilization of sodium chloride can cause a part of nickel and iron oxides to be volatile; (2) ferric and nickel chlorides, which generated by the way of interactions, volatilize at high temperature and then they are oxidized to form oxides^34^.

### Analyses of reduction roasted ore

**Figure 13:**
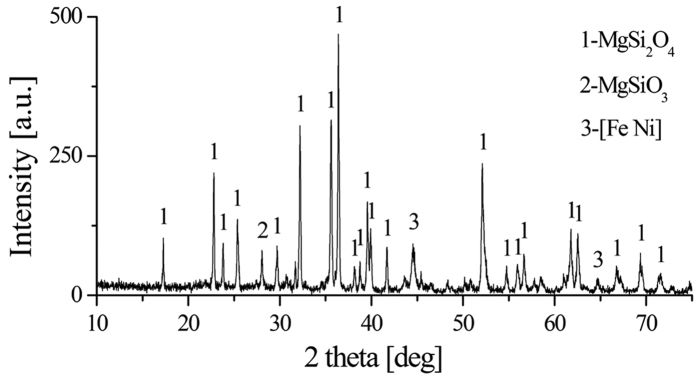
XRD pattern of reduction roasted ore.

**Figure 14:**
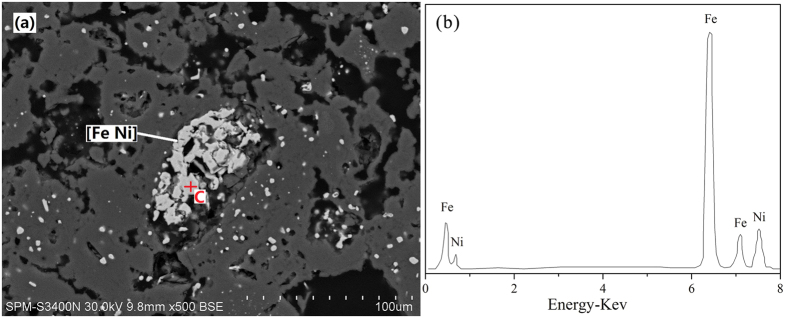
SEM/EDS analyses of chloridization and reduction roasted ore.

To further investigate the phase transformation of laterite ore upon roasting reduction as a result of drying, the XRD pattern of roasted ores in the presence of 10 wt.% sodium chloride under the conditions (raw ore dried at 650 °C, roasting temperature 1200 °C, roasting duration 20 min, 8 wt.% coal dosage) is analyzed in Fig. 13. In general, the forsterite [Mg_2_SiO_4_], and kamacite [Fe Ni] are the major phases, small quantities of enstatite [MgSiO_3_] is also found. The nickel and iron existed in roasted ore in the form of kamacite, indicating that the aggregation of nickel and iron occurred during the roasting reduction process. Furthermore, the SEM image (Fig. 14) shows that the roasted ores have a loose microstructure and the bright white particles, which analyzed by EDS, are Ni-Fe alloy. The metallic particles with a gain size about 80 μm, aggregating around the pores within forsterite. The result is in line with the Fig. 10d, demonstrating that the iron nickel oxides formed NiFe_2_O_4_ before reduced by reductant. The formation of large ferronickel particles is beneficial for exacting them from gangue by magnetic separation. This finding supports the previous results shown in Fig. 1 that the both recoveries of iron and nickel are improved with the drying of raw ore.

**Figure 15:**
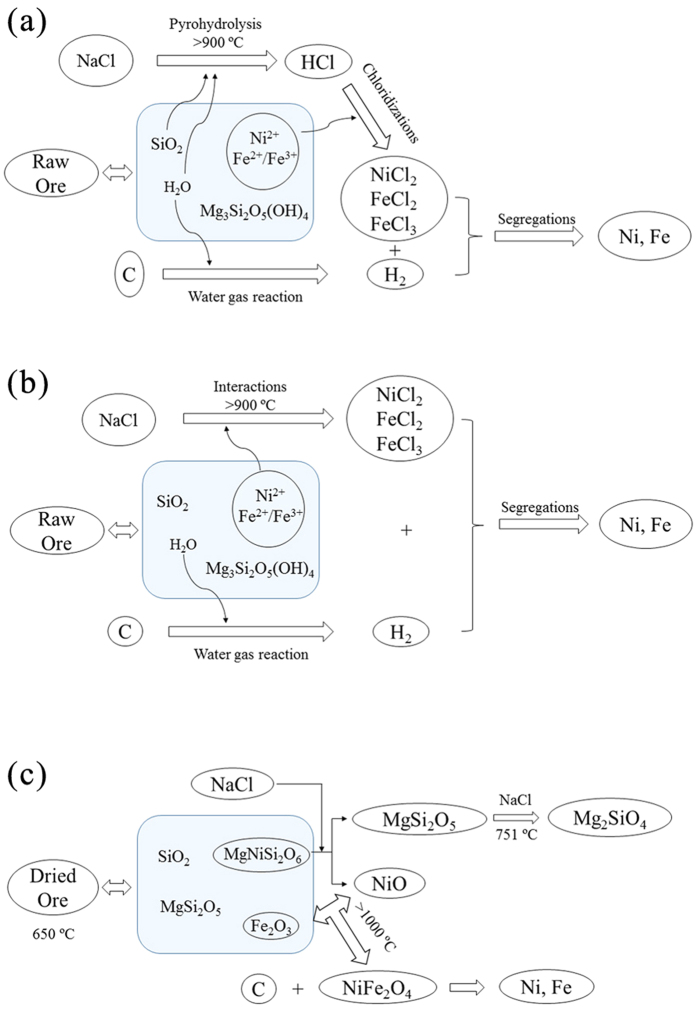
Type of action of NaCl in chloridization and reduction roasting process.

According to the above analyses, the ways of sodium chloride promote the reduction of ore are summarized in Fig. 15. In the presence of moisture, the iron and nickel oxides are chloridized by hydrogen chloride, which generated by pyrohydrolysis of sodium chloride; moreover, interactions between metallic oxides and sodium chloride are also a way for chlorination. In the absence of moisture, the sodium chloride existed in ore could make the nickel oxide isolate from serpentine, the structure of olivine is destroyed lead to aggregation of iron oxides. Furthermore, due to the change of the occurrence state of iron and nickel, the NiFe_2_O_4_ would be generated more easily with the action of sodium chloride at high temperature.

## Conclusions

The mechanism of sodium chloride in promoting reduction of high-magnesium low-nickel oxide ore in the absence of moisture is investigated. After drying at 650 °C, the moisture within HL oxide ore is completely removed and the specific surface area decreases from 74.042 to 50.671 m^2^/g. The recoveries of nickel and iron in ferronickel concentrate increase with increasing drying temperature of raw ore. Metallic oxide within ore can be chloridized by the interactions with sodium chloride, nonetheless, it would not be the main route for promoting ore reduction because of the kinetic conditions. In the absence of sodium chloride, the NiO would be fixed by serpentine, which suppressed NiO reduction. Sodium chloride could promote the phase transformation of serpentine into olivine and make the NiO isolate from serpentine; the NiO reacts with Fe_2_O_3_ to form NiFe_2_O_4_, this reaction promotes the aggregation of ferronickel. The SEM results demonstrate that the structure of olivine in the presence of sodium chloride would be destroyed at high temperature (>850 °C), and iron oxides would aggregate at the crack.

## Additional Information

**How to cite this article**: Zhou, S. *et al.* Mechanism of sodium chloride in promoting reduction of high-magnesium low-nickel oxide ore. *Sci. Rep.* **6**, 29061; doi: 10.1038/srep29061 (2016).

## Figures and Tables

**Table 1:** Chemical analysis of the laterite ore sample.

Component	Fe (total)	Ni	Co	MgO	SiO_2_	Al_2_O_3_	CaO	S	Mn
Content wt.%	9.67	0.82	0.033	31.49	37.37	1.89	0.033	0.01	0.083
